# 3D Printed Graphene-PLA Scaffolds Promote Cell Alignment and Differentiation

**DOI:** 10.3390/ijms23031736

**Published:** 2022-02-03

**Authors:** Matteo Gasparotto, Pietro Bellet, Giorgia Scapin, Rebecca Busetto, Chiara Rampazzo, Libero Vitiello, Dhvanit Indravadan Shah, Francesco Filippini

**Affiliations:** 1 Synthetic Biology and Biotechnology Unit, Department of Biology, University of Padua, 35131 Padua, Italy; matteo.gasparotto.1@phd.unipd.it (M.G.); pietro.bellet@studenti.unipd.it (P.B.); rebecca.busetto@studenti.unipd.it (R.B.); 2 Garuda Therapeutics, Cambridge, MA 02142, USA; dhvanit.shah@garudatx.com; 3 Department of Biology, University of Padua, 35131 Padua, Italy; chiara.rampazzo.1@unipd.it (C.R.); libero.vitiello@unipd.it (L.V.); 4 Interuniversity Institute of Myology (IIM), Administrative headquarters University of Perugia, Piazza Lucio Severi 1, 06132 Perugia, Italy; 5 Inter-Departmental Research Center for Myology (CIR-Myo), University of Padua, 35131 Padua, Italy

**Keywords:** graphene, G+, PLA, Grafylon, scaffold, neuronal differentiation, stem cells, 3D printing, regenerative medicine

## Abstract

Traumas and chronic damages can hamper the regenerative power of nervous, muscle, and connective tissues. Tissue engineering approaches are promising therapeutic tools, aiming to develop reliable, reproducible, and economically affordable synthetic scaffolds which could provide sufficient biomimetic cues to promote the desired cell behaviour without triggering graft rejection and transplant failure. Here, we used 3D-printing to develop 3D-printed scaffolds based on either PLA or graphene@PLA with a defined pattern. Multiple regeneration strategies require a specific orientation of implanted and recruited cells to perform their function correctly. We tested our scaffolds with induced pluripotent stem cells (iPSC), neuronal-like cells, immortalised fibroblasts and myoblasts. Our results demonstrated that the specific “lines and ridges” 100 µm-scaffold topography is sufficient to promote myoblast and fibroblast cell alignment and orient neurites along with the scaffolds line pattern. Conversely, graphene is critical to promote cells differentiation, as seen by the iPSC commitment to neuroectoderm, and myoblast fusions into multinuclear myotubes achieved by the 100 µm scaffolds containing graphene. This work shows the development of a reliable and economical 3D-printed scaffold with the potential of being used in multiple tissue engineering applications and elucidates how scaffold micro-topography and graphene properties synergistically control cell differentiation.

## 1. Introduction

It is the well-known features of the tissue environment, such as the mechanical, electrical, and molecular cues, which play a pivotal role in regulating cell differentiation, proliferation, shape and orientation. Therefore, the ability of scaffolds to mimic tissue environmental stimuli is crucial in regenerative medicine and tissue engineering projects [1]. Decellularised tissues from animal donors or human cadavers are promising ‘physiological’ scaffolds encapsulating the natural composition and the three-dimensional organisation of the extracellular matrix (ECM) [2]; however, antigens from such scaffolds may elicit adverse immune responses leading to transplant failure [3], and the batch-to-batch variability would impair the reliability of protocols and results [4]. To alleviate these issues, in recent years the focus has switched to the design and development of synthetic biocompatible polymers and nanocomposites which represent a much more attractive option for drug delivery, biosensors and tissue engineering applications [5]. Among them, Poly-L-Lactic Acid (PLA) has proven reliable for tissue engineering manufacturing, as it shows low immunogenicity, batch variation and easy modulation of its physical and biochemical features by blending with different nanofillers [6].

PLA-based scaffolds are self-standing and can be used for cell growth and differentiation. In vivo, bare PLA is degraded by non-enzymatic hydrolysis of esther bonds [7]. Although it is fully reabsorbed, degradation rate is very slow, and PLA residues can be detected up to 5.5 years in the human body even after it loses its functions as biomaterial [8]. Such features make PLA scaffolds suitable for planned reabsorption [9] and are therefore the preferred base materials for the fabrication of artificial tissue or scaffolds for tissue regeneration [10].

Since cells have micro and nanoscale sensitivity and are able to respond to an extracellular environment presenting a variety of spatially defined cues in the sub-micron to micron scale, the incorporation of nanomaterial in biocompatible polymers greatly improves the scaffold performance by mimicking the multiple environmental stimuli of the chosen tissue [4,11]. Among the plethora of nanomaterials available, graphene and its derivatives are attractive candidates for developing tissue engineering scaffolds due to their tuneable electrical conductivity, excellent mechanical properties, biocompatibility, chemically modifiable surface, and nanoscale dimension matching cell surface receptors and extracellular matrix (ECM) nanotopography [12]. Graphene consists of a two-dimensional monolayer of sp^2^ hybridised carbon atoms bonded in a hexagonal lattice [13]. Although its physico-chemical properties make it an attractive material in several fields [14,15,16], its use for biological applications has been shown to have a dose- and time-dependent toxicity [17,18,19]. Since its first discovery, several derivatives, including graphene oxide (GO) and reduced graphene oxide (rGO) have been developed to both reduce fabrication costs and modulate physico-chemical properties [20,21,22]. Particularly, properties of graphene-based nanomaterials (GBN) are strongly influenced by several factors, including shape, size, lateral dimension, surface chemistry and defect density. GO and rGO based nanomaterials are less hydrophobic than graphene-based ones, thus showing reduced aggregation and improved biocompatibility; however, toxicity of bare GBN is still a matter of concern. For instance, cytotoxicity of GO flakes used for neuronal differentiation of stem cells has been found to be strongly dependent on flake dimensionality, whereas surface roughness was responsible for better cell attachment and proliferation [23]. Surfaces with large flakes of GO or rGO are the most biocompatible for mesenchymal stem cells propagation and do not affect their proliferation and survival [24]. It has been shown than incorporation of GBN into polymeric matrices can further reduce their toxicity and, at the same time, improve properties of the dispersing matrix [25].

In neural regenerative projects, incorporation of graphene in polymeric scaffolds endows them with nanoroughness, which contributes to cell anchoring and modulation of cell morphology, and allows the establishment of tight contact with the growth cone that guides the spreading of developing neurites [26]. Since for regenerating axons it is critical to reach their correct targets, the development of scaffolds incorporating patterns of graphene could provide the physical guidance cues to allow neurite elongation facilitating nerve formation [27]. In addition, neuronal differentiation of stem cells or precursor cells is boosted by electrically conductive scaffolds mimetic of the neural tissue electroactive properties [12,28,29]. As graphene is electrically conductive and its conductivity is stable in biological environments, its incorporation in polymeric scaffolds can reduce the polymer electrical resistance and offer a permissive environment for neuronal differentiation.

Electrical and nanotopographical stimuli are not only important for the neuronal cell physiology but also play a vital role in multiple cellular process like myoblast fusion and wound healing. After skeletal muscle injury, myoblast precursors first migrate to the damaged site, then they align longitudinally, and finally they fuse their membranes to form multinuclear myotubes [30]. Since such geometrical directionality has a close relationship with function, the simulation of the ECM natural topography is a critical factor in the generation of muscle tissue engineering scaffolds [31]. Like myoblasts, fibroblasts are sensitive to topographical cues that play a critical role in the wound healing process. Fibroblast alignment allows deposition of highly anisotropic ECM and collagen fibres which support the wound healing process whilst reducing the scar tissue formation [32]. By being strongly dependent on ionic channel activation and Ca^2+^ concentration [30], myoblast fusion is facilitated by electrically conductive scaffolds. Likewise, the wound regeneration process is accelerated by conductive materials supporting the transmission of endogenous/exogenous electrical stimuli [33]. Therefore, scaffolds bearing graphene patterns have great potential in providing contact guidance to alignment, ECM protein-like nanofeatures to stimulate intracellular-responsive pathways, and electrically conductive properties which finally enhance myoblast membrane fusion [34,35] and wound healing [36,37,38,39].

Although graphene-based nanocomposite scaffolds can modulate cell fate, even small variations in their composition can have unpredictable effects on cellular response [40]. In recent years, fused deposition manufacturing (FDM) 3D-printing has emerged as a cost-effective, reliable, and reproducible tool to rapidly generate scaffold for cell growth with desired micro-topographical features. The main drawback of FDM is the inherent presence of wrinkles on the surface of objects due to the side-by-side deposition of extruded material; however, even those micro-topographic imperfections (i.e., grooves, pillars, and ridges) have been shown to influence migration and orientation of various cell lines [41].

In this work, we exploited FDM to develop 3D printed scaffolds based on either PLA or graphene@PLA with defined patterns and we tested them with different cell types to assess how scaffolds influence cell behaviour. Such scaffolds resulted to be biocompatible and to promote the alignment of neuronal, fibroblast and myoblast cells due to their 3D printed topographies. In addition, the scaffolds containing the graphene could stimulate the differentiation of induced pluripotent stem cells (iPSCs) to neuroectodermal precursors and promote the myoblast membrane fusion to generate multinuclear myotubes. In summary, we obtained state-of-the-art and cost-effective scaffolds with the potential for being applied in multiple regenerative medicine applications. Such scaffolds intrinsic properties can directly stimulate the desired cell behaviour, without addition of any exogenous factor.

## 2. Results

### 2.1. 3D Printing

Scaffold design and printing parameters were optimised to obtain a regular pattern of creases at increasing distance, to test whether an increase in pattern spacing could influence cell alignment and differentiation. Ultimately, we opted for two series of micropatterned scaffolds that correspond to two different printing techniques: (i) 100 µm-spaced scaffolds (100 µm) and (ii) 400 µm-spaced scaffolds (400 µm). The 100 µm series was designed so that neighbouring filament depositions were at a distance comparable with that of a cell in its larger dimension. These scaffolds were built vertically from the printing table to take advantage of the higher resolution of the z printing axis, though alterations on the printing bed led to a more consistent ~100 µm pattern. Such increase in resolution also involves an increase in roughness, as single lines of filament are extruded on top of each other making the scaffold surface not completely even. To fix this predicament, the 400 µm series took advantage of the smooth surface of the build table itself to yield objects with a flat surface. Scaffolds were printed upside-down: a single line of material was deposited back-and-forth on the xy plane, spaced apart by 400 µm (much higher than the average diameter of a cell soma). Then, the build plate was lowered, and fused filament was poured in larger quantities, higher temperature, and lower speed along a complementary path, so it could expand and completely fill in the empty spaces. Therefore, ridges form only in near proximity of the initial path: this allows to obtain flat surfaces with wrinkles roughly every 400 µm.

**Figure 1:**
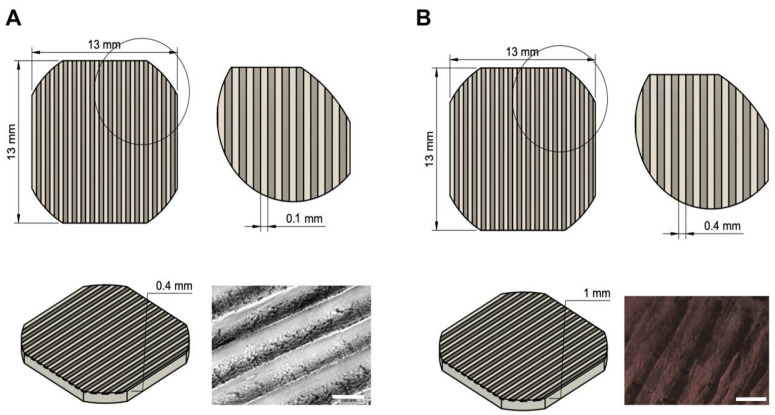
Frontal, lateral and axonometric views of scaffold design. (**A**) 100 µm series (**B**) 400 µm series. Scalebar of the fourth image of each panel is 100 µm and 400 µm, respectively.

Using the methods described above, we were able to create micropatterned scaffolds made entirely of PLA or graphene@PLA, as seen in Figure 1.

### 2.2. Cell Viability and Proliferation

**Figure 2:**
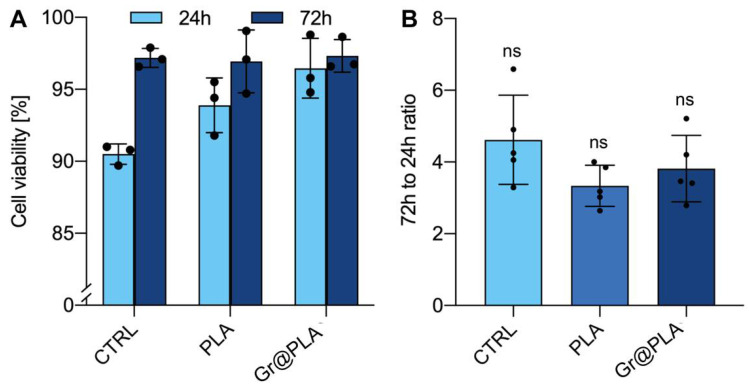
Effects of bare Poly-L-lactic acid (PLA) or graphene@PLA (Gr@PLA) on SH-SY5Y. (**A**) Cell viability at either 24 or 72 h after cell seeding. Lower values at 24h are due to post-detachment stress. (**B**) Cell proliferation expressed as ratio between number of cells at 72h and 24h after seeding. Scaffolds do not significantly interfere with cell proliferation. All data represent the mean ± SD of at least three independent experiments. Statistical significance was determined with one-way ANOVA with Tukey’s correction.

Carbon-based nanofillers, including graphene, have been reported to be biocompatible at concentrations up to 3–5% in either PLA [42] or other matrices [43] and although graphene concentration in graphene@PLA is as low as roughly 1%, i.e., similar to already validated, graphene@PLA flat scaffolds [29,40], we tested viability and proliferation of cells cultured onto both graphene@PLA and corresponding pure PLA filaments to exclude that any negative effects from eventual impurities impaired biocompatibility. Figure 2A shows that viability of SH-SY5Y cells at 24 h (Day 1) and 72 h (Day 3) after seeding onto graphene@PLA and corresponding pure PLA scaffolds is not significantly different from cells seeded onto plates used as control. Viability ranges between 90 and 100%, and slightly lower values at Day 1 are known to depend on post-detachment stress [42].

Once cytotoxicity was excluded, we checked eventual effects on cell proliferation, because exogenous materials eventually stimulating cell division are potentially tumorigenic. Figure 2B shows that proliferation of cells grown on the scaffolds is never significantly different from control, suggesting scaffolds do not alter cell proliferation.

### 2.3. 100 µm Graphene@PLA Scaffolds Promote Neuronal Commitment and Neurite Sprouting whilst Directing Neurite Elongation

**Figure 3:**
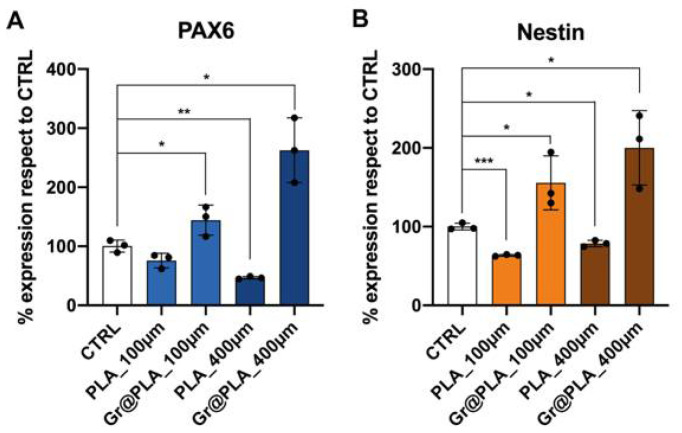
Neuroectodermal commitment of iPSCs grown onto 3D-printed scaffolds. (**A**) PAX6 and (**B**) Nestin expression increases when cells are cultured on graphene@PLA (Gr@PLA) scaffolds. All data represents the mean ± SD of at least three independent experiments. Statistical significance was determined with a two tailed *t*-test. Significance at *p* < 0.05 (*), *p* < 0.01 (**) and *p* < 0.001 (***) between samples is reported. CTRL condition consists of cells seeded onto common 24-well plates.

To test whether the bio-printed scaffolds could promote the neuronal differentiation of pluripotent stem cells, we cultured iPSCs onto the scaffolds with ectoderm-inducing media and we performed gene and protein expression analyses. As shown in Figure 3A,B graphene-containing scaffolds promote the expression of the transcription factor PAX6, which is the master regulator of the neuroectoderm specification [44] and of Nestin, an intermediate filament protein mainly expressed by neural progenitor cells with an important role in cellular remodelling [45]; however, cells did not seem to follow the spatial organisation of the scaffold (not shown), possibly because they are still too immature and not yet extending neurites.

Therefore, to investigate if the scaffold pattern could trigger the neurite sprouting and orient their elongation, we used the neuronal-like cell line SH-SY5Y. This cell line is characterised by neuroblast-like, non-polarised cell bodies with few truncated processes. Moreover, cells grow in clusters and express immature neuronal markers, which make them resemble immature catecholaminergic neurons [46]. Upon differentiation, SH-SY5Y cells express mature neuronal markers, such as the neurotrophin receptor (TrKB) and increase their neurite length and number [47,48].

**Figure 4:**
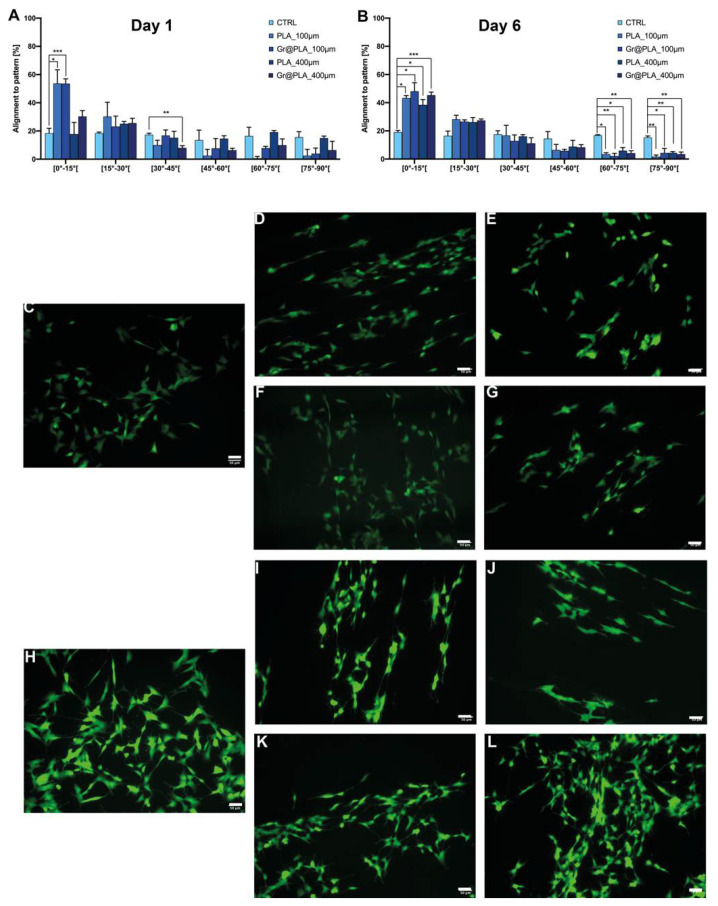
Alignment of SH-SY5Y cells to scaffold patterning. Values are at (**A**) day 1 and (**B**) day 6 from cell seeding. Cell orientation is influenced by scaffold patterning independently of its composition. CTRL condition consists of cells seeded onto common 24-well plates. All data represents the mean ± SD of at least three independent experiments. Statistical significance was determined with two-way ANOVA with Dunnet’s correction; significance at *p* < 0.05 (*), *p* < 0.01 (**) and *p* < 0.001 (***) between samples is reported, (**C**–**L**) representative fields of cells grown onto scaffolds. (**C**) Day 1 control, (**D**) Day 1 PLA_100 µm, (**E**) Day 1 PLA 400 µm, (**F**) Day 1 Gr@PLA_100 µm, (**G**) Day 1 Gr@PLA_400 µm, (**H**) Day 6 control, (**I**) Day 6 PLA_100 µm, (**J**) Day 6 PLA 400 µm, (**K**) Day 6 Gr@PLA_100 µm, (**L**) Day 6 Gr@PLA_400 µm. Scalebar 50 µm.

Although scaffolds did not result in a significant increase in neurite length or number (Supplementary Figure S1), they were able to strongly influence cell cluster formation. Specifically, we analysed the contact guidance of SH-SY5Y by considering the angle between the pattern and the trajectory of processes and we addressed the alignment in terms of percentage of neurites within a specific angle range on the total number of neurites. As shown in Figure 4, SH-SY5Y better align on 100 µm scaffolds compared to 400 µm. This is probably due to the different dimensionality of the two micropatterns. The 400 µm series has 400 µm-wide flat surfaces divided by creases: since SH-SY5Y neurites reach only ~100 µm in length, smooth surfaces are vast enough to be sensed as non-patterned and neurites sprout randomly (~20% in each angle range). On the other hand, 100 µm scaffolds are designed with a denser pattern, so non-aligned neurites forcefully get into contact with it, resulting in a higher percentage of orienteered neurites.

Together, whilst the graphene presence in the scaffold boosts the iPSC commitment to neuroectoderm, the specific 100 µm printing pattern orient the neurite elongation along the scaffold aligned features.

### 2.4. Contact Guidance Is Influenced by 3D-Printing Method and Promotes Myoblast Fusion into Multinuclear Myotubes When Combined with Graphene Cues

Cell alignment is critical for the fibroblast deposition of anisotropic matrix favouring wound healing and for the myoblast fusion into multinuclear myotubes. Given the ability of our 3D printed scaffolds to affect neurite orientation, we hypothesised that scaffold composition and pattern could also affect contact guidance of fibroblasts and myoblast leading to an aligned cell organisation. We tested contact guidance as the percentage of cells with a specific angle with respect to scaffold pattern. We analysed cell morphology on the scaffold at 48 h and 168 h after cell seeding for immortalised fibroblasts and at 72 h after cell seeding for immortalised myoblasts.

**Figure 5:**
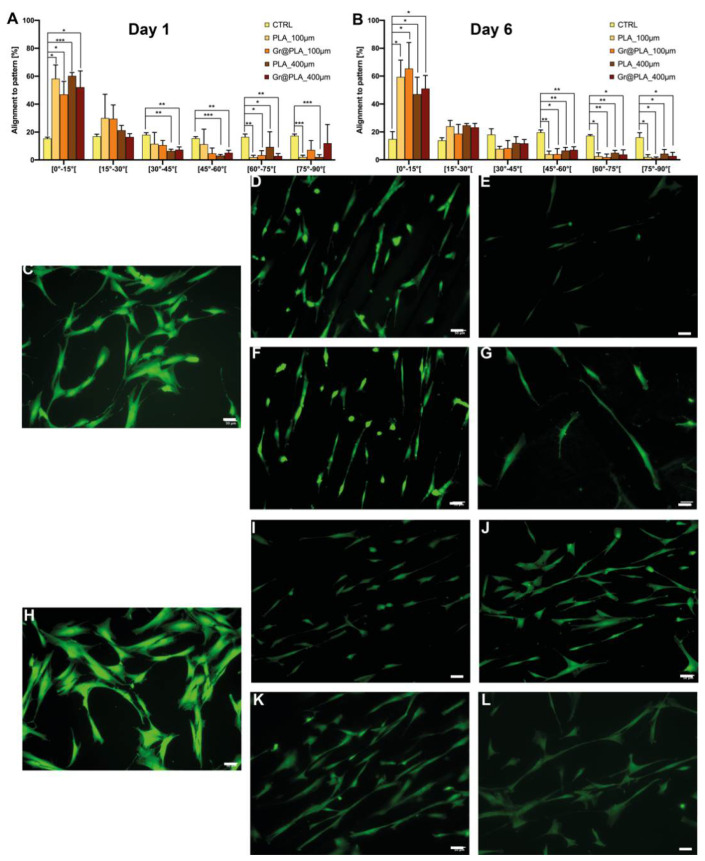
Alignment of hTERT-immortalised fibroblasts to scaffold patterning. Values are at (**A**) day 1 and (**B**) day 6 from cell seeding. Cell growth is influenced by scaffold patterning independently of its composition. CTRL condition consists of cells seeded onto common 24-well plates. All data represent the mean ± SD of at least three independent experiments. Statistical significance was determined with two-way ANOVA with Dunnet’s correction; significance at *p* < 0.05 (*), *p* < 0.01 (**) and *p* < 0.001 (***) between samples is reported, (**C**–**L**) representative fields of cells grown onto scaffolds. (**C**) Day 1 control, (**D**) Day 1 PLA_100 µm, (**E**) Day 1 PLA 400 µm, (**F**) Day 1 Gr@PLA_100 µm, (**G**) Day 1 Gr@PLA_400 µm, (**H**) Day 6 control, (**I**) Day 6 PLA_100 µm, (**J**) Day 6 PLA 400 µm, (**K**) Day 6 Gr@PLA_100 µm, (**L**) Day 6 Gr@PLA_400 µm. Scalebar 50 µm.

As shown in Figure 5, hTERT-immortalised fibroblasts seeded onto scaffolds were aligned to the printing axis, whereas control cells displayed random orientation.

This was expected, as fibroblasts are known to be highly responsive to topographical cues. 100 µm scaffolds have an increased density of topographical stimuli; thus, we were expecting a higher percentage of aligned cells on those scaffolds compared to 400 µm. However, this was not the case, as alignment on 400 µm and 100 µm at day 6 is not meaningfully different. Intriguingly, Miyoshi and co-workers [49] found fibroblasts to migrate and align along the longitudinal axis of micropatterned surfaces, therefore, the vast majority of cells moved near scaffold ridges and experienced contact guidance, independently from scaffold pattern dimensionality and graphene presence.

**Figure 6:**
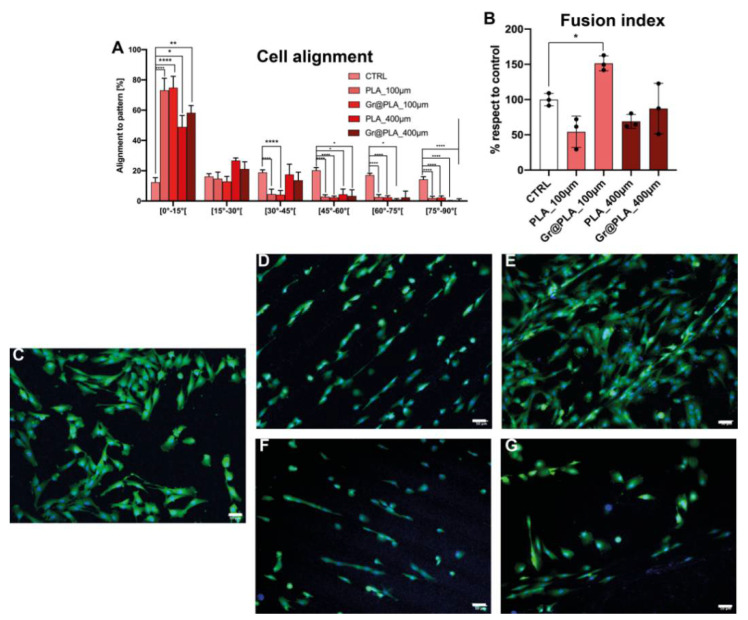
Effects of 3D-printed scaffolds on myoblasts. (**A**) Myoblasts alignment to scaffold pattern is strongly influenced by scaffold dimensionality, however it is independent from scaffold composition. (**B**) Fusion index is strongly increased by the combined effects of graphene and patterning. CTRL condition consists of cells seeded onto common 24-well plates. All data represents the mean ± SD of at least three independent experiments. Statistical significance of panel (**A**) with two-way ANOVA with Dunnet’s correction, whereas that of panel (**B**) was determined with one-way ANOVA with Tukey’s correction; significance at *p* < 0.05 (*), *p* < 0.01 (**), *p* < 0.001(***) and *p* < 0.0001 (****) between samples is reported, (**C**–**G**) representative fields of cells grown onto scaffolds. (**C**) control, (**D**) PLA_100 µm, (**E**) PLA 400 µm, (**F**) Gr@PLA_100 µm, (**G**) Gr@PLA_400 µm.

We recently reported reduced graphene oxide (rGO) PLA composite scaffolds to be able to increase the expression of pro-myogenic markers on human circulating multipotent cells [40]; however, manually cast scaffolds were unable to increase fusion index of primary myoblasts in culture. We then wondered if the improved control over surface roughness and pattern of 100 µm and 400 µm printed scaffold could solve this issue. As shown in Figure 6A, cell alignment ability seems to be independent of the scaffold composition but rather on the topographies generated by the 3D printing process. Conversely, when analysing the ability of cells to fuse their membranes, we found that the 100 µm-graphene@PLA scaffolds almost doubled the fusion index of control cells, whilst 400 µm- graphene@PLA scaffolds and PLA-only scaffold were unable to improve the cell membrane fusion capacity (Figure 6B).

Altogether, such data suggest that contact guidance and alignment are purely regulated by the scaffold features provided by the 3D-printing process, whilst cell behaviours like membrane fusion are controlled by a combination of 3D-printed cues and graphene incorporation.

## 3. Discussion

Although nervous, muscle, and connective tissues can partially regenerate after injury, in chronic damages or after traumas, their endogenous self-regeneration is impaired and, consequently, tissue engineering approaches are promising therapeutic tools; however, despite the advances in microfabrication techniques, tissue engineering applications are still struggling to develop immune-compatible biomaterials, retaining the features of the natural tissue of interest. Therefore, it is critical to develop reliable, reproducible, and economically affordable synthetic scaffolds which could provide sufficient biomimetic cues to promote the desired cell behaviour without triggering graft rejection and transplant failure. Poly-L-lactic acid-based scaffolds demonstrated high versatility in promoting cell growth and differentiation. We previously found that rGO@PLA based scaffolds could modulate cell commitment toward the neuronal or muscle fate [40]; however, manual casting of scaffolds proved unreliable and expensive. Here, we took advantage of cost-effective fused deposition manufacturing 3D-printing technology and commercially available nanocomposite filaments to design biocompatible scaffolds which can be used as a platform for different tissue engineering applications. We found that both PLA and graphene@PLA filaments do not significantly alter cell vitality as viability was found to be above 90%. Moreover, a 3- to 5-fold increase in cell number between 24 h and 72 h after seeding was found in all condition tested and is compatible with the 27 h doubling time of the SH-SY5Y cell line [46], indicating scaffolds do not alter cell proliferation. When iPSCs were seeded onto scaffolds, we found graphene could increase expression of Pax6 and Nestin, independently on scaffold micro-topography, suggesting commitment toward the neuronal lineage. Conversely, no condition tested was found to induce an enhancement in neurite sprouting or elongation of the more mature cell line SH-SY5Y. As SH-SY5Y cell differentiation has been associated with a decrease in cell proliferation, this is in agreement with reported observations on proliferation rate [50]. Moreover, SH-SY5Y growth appears strongly oriented toward scaffold topography. Albeit cells are strongly oriented both on day 1 and day 6, differences observed between the 100 µm and 400 µm class might be due to cell migration timing. Evidence reported in the literature suggests migration of proliferating SH-SY5Y is poor and substrate dependent [51,52]. 3D printed scaffolds proved to influence cells outside the neuronal lineage, as micro-topographical features influenced orientation of hTERT-immortalised fibroblasts and myoblasts. Strikingly, myoblasts seeded onto 100 µm scaffolds containing graphene, showed an increase in fusion rate into multinuclear cells, indicating patterning and graphene are both required to favour myotube formation. In conclusion, here we show the development of a reliable and economical FDM printed scaffold with the potential of being used in multiple tissue engineering applications, and we elucidate how micro-topographies provided by the 3D printed line pattern and the nano-topographies/conductive properties provided by the graphene incorporation synergistically control cell differentiation.

## 4. Materials and Methods

### 4.1. 3D-Printing

Poly-L-lactic acid (PLA) and graphene@PLA (GRAFYLON^®^ 3D) ø 1.75 mm filaments were purchased from FILOALFA, Ozzero, Italy. GRAFYLON^®^ 3D consists of G+ Graphene Plus (Directa Plus, Lomazzo, Italy) dispersed in pure PLA. G+ Graphene Plus is made up of pristine graphene nanoplatelets (GNPs), which are obtained by purely physical treatments of natural graphite, thus avoiding any chemical treatments with organic solvents or acids, and just exploiting water, temperature and pressure to reduce the graphite thickness to the nanometric level. G+ Graphene Plus GNPs have a lateral dimension in the micrometer range, whilst the thickness is in the nanometer scale (https://graphene-plus.com/ accessed on 19 January 2022). The 3D-printable scaffolds were designed using Fusion 360 (v2.0.9719, Autodesk, San Rafael, CA 94903, USA), whilst 100 µm-micropatterned scaffolds were developed as 50 × 50 × 15 mm hollowed cubes with 1.0 mm wall width and presented regularly alternated materials every 50 µm. Final scaffolds were cut from the cube into round-edged squares of approximately 13.0 × 13.0 mm and 1.0 mm in height, well suited for 24-well plates. To obtain scaffolds with a patterning much higher than average cell length, 400 µm-patterned scaffolds were designed directly as 13.0 × 13.0 × 1.0 mm round-edged squares with a 400 µm-wide pattern. STL files were imported in Simplify3D (v4.1.2, Cincinnati, OH 45241, USA) and slicing presets were configured. Layer height and nozzle temperature varied based on the type of support and material: 100 µm scaffold series was printed with 0.050 mm layer height, 100% infill at 195 °C for both PLA and graphene@PLA, whereas 400 µm series was printed with 0.1 mm layer height, 100% infill, 195 °C for graphene@PLA and 0.2 mm layer height, 100% infill, 215 °C for PLA. To improve first layer adhesion, the printing plate was coated with a saturated solution of sucrose in water. Specifically, the sucrose solution was dispersed onto the glass-slide printing plane. The printing plane was then heated to 40 °C and water was left to evaporate to create a sticky film over the glass. Models were printed by fused deposition manufacturing (FDM) with a NG dual 3D printer (Sharebot, Nibionno, Italy). After printing, scaffolds were sterilised under UV-light, 30 min on all sides after a 30 min wash in 75% ethanol. Pure PLA and graphene@PLA 3D-printed scaffolds were pre-incubated for 3h in DMEM/F-12 10% FBS (growth medium).

### 4.2. SH-SY5Y Cell Culture and Differentiation

Exponentially growing human neuroblastoma-derived SH-SY5Y cells were cultured with Dulbecco’s Modified Eagle Medium/Nutrient Mixture F-12, GlutaMAX™ supplement (DMEM/F-12, Gibco™, Fisher Scientific Italia, Rodano, Italy), enriched with 10% heat-inactivated foetal bovine serum (FBS, Euroclone) and 25 μg/mL gentamicin (Sigma-Aldrich, St. Louis, MO, USA), in a humidified atmosphere of 5% of CO_2_ in air at 37 °C. Cells were split into 25 cm^2^ flasks (Sarstedt, Nümbrecht, Germany) every 2 days. Cell differentiation was induced by treating cells with all-trans-retinoic acid (RA, Sigma-Aldrich) at 10mM concentration and lowering the FBS in the culture medium to 2% (differentiation medium) 24 h after seeding. In undifferentiated control samples, Dimethylsulfoxide (in which RA is dissolved) was added as the equivalent amount.

### 4.3. Immortalised Fibroblast Cell Culture

hTERT-immortalised fibroblasts were cultured in high glucose Dulbecco’s Modified Eagle Medium, GlutaMAX™ and pyruvate supplement (DMEM, Gibco™) enriched with 10% FBS, MEM non-essential amino acids (NEAA, Gibco™), 50 U/mL penicillin and 50 µg/mL streptomycin (Pen/Strep, Gibco™) in a humidified atmosphere of 5% of CO_2_ in air at 37 °C. Cells were split into 75 cm^2^ vented flasks (Sarstedt, Nümbrecht, Germany) every 3 days.

### 4.4. Immortalised Myoblasts Cell Culture

Immortalised human myoblasts, obtained by double transduction with hTERT and cdk4, were kindly supplied by the Institut de Myologie (Pitié-Salpétrière Hospital, Paris, France). These myoblasts (wt AB1190) were cultured with a growth medium containing F12 supplemented with 20% FBS (Invitrogen Life Technologies, Waltham, MA, USA), 25 µg/mL fetuin (Invitrogen Life Technologies), 5 ng/mL hEGF (ImmunoTools GmbH, Friesoylthe, Germany), 0.5 ng/mL bFGF (ImmunoTools GmbH), 5 µg/mL insulin (Sigma-Aldrich), and 0.2 µg/mL dexamethasone (Sigma-Aldrich).

### 4.5. Cell Proliferation Assay

Cell proliferation was measured using the resazurin reduction assay. The assay is based on the reduction of the non-fluorescent indicator dye resazurin to the highly fluorescent resorufin (Ex 569 nm, Em 590 nm) by viable cells. Cells were seeded onto scaffolds or 24-well plates as control (time 0) and cell number was evaluated at 24 h and 72 h after seeding. At each time point, the culture medium was replaced by resazurin solution (resazurin, 15 μg/mL in growth medium; Sigma-Aldrich) and cells were incubated for 4 h in the dark at 37 °C, 5% CO_2_. Absorbance at 590 nm was detected using a Fluoroskan Ascent fluorometer (Fisher Scientific Italia, Rodano, Italy) and background values from blank samples were subtracted. To infer the number of cells in each sample, a titration curve was obtained by performing the assay on a known number of cells seeded onto control gelatin/poly-*L*-lysine coated wells. The number of cells in each condition tested was determined by linear regression from the titration curve and cell proliferation was determined as the ratio of the number of cells 72 h and 24 h after seeding.

### 4.6. Cell Viability Assay

Cell viability was assessed with CytoTox-ONE™ Homogeneous Membrane Integrity Assay, (Promega Italia, Milano, Italy), which is based on the estimation of the LDH released by dead cells. According to manufacturer protocol, cells were grown onto scaffolds for 24h or 72h. At each time point, an equal volume of CytoTox-ONE™ Reagent was added and samples were incubated for 10 min. Reaction was then stopped with blocking solution and fluorescence at 590 nm was detected using a Fluoroskan Ascent fluorometer (Fisher Scientific Italia). A negative (medium without cells) and positive (cells lysed with Promega Lysis Solution) control were used to determine blank and maximal LDH release. Toxicity was then evaluated using:

$$Percent\;Cytotoxicity=100\cdot \frac{Sample-Blank}{Maximum\;LDH\;Release-Blank}$$

### 4.7. Differentiation and Contact Guidance Analysis

Cells were seeded onto different types of 3D-printed scaffolds and morphology was evaluated at 24 h (Day 1) and 168 h (Day 6) (SH-SY5Y cells and immortalised fibroblasts) or 72 h (Day 3, myoblasts) after seeding. Based on each line proliferation rate, a different number of cells was seeded for observation at day 1 and 6. Specifically, proliferative SH-SY5Y were seeded 35 k/well for Day 1 and 20 k/well for Day 6. Fibroblasts were seeded 20 k/well for Day 1 and 10 k/well for Day 6. Finally, myoblasts were seeded onto scaffolds and gelatin-coated control wells at 25 k cells/well density. Culture medium was refreshed every 2 days.

Cells were visualised after incubation with calcein acetoxymethyl ester (Calcein-AM, Biotium, Fremont, CA 94538, USA), 1 µM in Hank’s Balanced Salt Solution (HBSS, Gibco™, Fisher Scientific Italia) and 10 µg/mL Hoechst 33258 (Invitrogen Life Technologies) for 30 min in the dark at 37 °C and 5% CO_2_. Medium was then replaced with fresh HBSS and cells were observed with a DMI4000 microscope (Leica, Wetzlar, Germany) at 10× magnification with a GFP and DAPI filter. Ten images per well were recorded; the first two fields were set to correspond to the centre of the well. Next, fields were then selected in the periphery of the well (N, NE, E, SE, S, SW, W, NW, in respect to the centre), so that images could be representative of the whole well. Images were evaluated with Fiji suite v. 2.3 [53]. Cells were counted by manually counting nuclei. Overall, between 1000 and 2000 cells were recorded per condition. SH-SY5Y differentiation was evaluated considering neurite outgrowth and elongation. Neurite length was measured using Fiji suite [53] by tracing the path of the neurite from the tip to the junction between the neurite and cell body. Processes were considered neurites when their length was longer than 50 µm and neuritogenic properties were analysed in terms of total number of neurites to cell ratio and their mean length as described in [54]. Contact guidance on neurite growth and fibroblast and myoblasts orientation was evaluated comparing the angle (0–90°) between the trajectories of neurites or cell bodies and the pattern. Cells were sorted into 15° classes, and data are presented as percentage of cells in a given class respect to the total number of cells. Cells or neurites were considered aligned if their angle did not exceed 15° respect to pattern angle. All experiments were performed in triplicate.

### 4.8. Fusion Index Analysis

Myoblasts were stained with 2 µM Calcein-AM (Biotium) in HBSS (Gibco™, Fisher Scientific Italia) and 10 µg/mL Hoechst 33258 (Invitrogen Life Technologies) for 45 min in dark at 37 °C and 5% CO_2_. Cells were visualised under a DM4000B fluorescent microscope (Leica) using GFP and DAPI filter. Fusion index, which describes the number of nuclei inside myotubes as a percentage of the total number of nuclei, was evaluated.

### 4.9. iPSC Culture and Neuroectodermal Induction

iPSCs (A18945, Thermofisher, Waltham, MA, USA) were cultured on a Matrigel substrate (Corning, Somerville, MA, USA) with mTeSR media (Stemcell Technologies, Vancouver, BC, Canada) and maintained at 37 °C, 5% CO_2_, 95% humidity. For neuroectodermal induction, cells were detached from the culture dish when 70% confluent with TripLE (Thermofisher) incubation for 5 min at 37 °C to obtain a single cell suspension. Next, 200K cells/well were seeded onto Matrigel coated wells or on scaffolds placed in a 24-well plate in mTeSR supplemented with 2uM of Y-27632 Dihydrochloride (Peprotech EC, London, UK). 24h after seeding, culture media was replaced with the ectodermal inducing media (R & D systems, Minneapolis, MI, USA) and cultured for additional 3 days. On day 4, cultures were stopped to perform RT-qPCR and immunofluorescence.

### 4.10. RT-qPCR

Media was removed from the wells, cells were incubated with TRIzol (Thermofisher) for 5 min at RT, and RNA extraction was performed using the RNeasy Micro Kit (Qiagen) following the manufacturer instruction. Next, cDNA synthesis was achieved by using the iScript cDNA Synthesis Kit (Bio-Rad, Hercules, CA, USA) following manufacturer instruction.

Gene expression was then analysed by qPCR. 1 ul of cDNA was used for each reaction that was performed using SYBR green (Quantabio, Beverly, MA, USA) in a final reaction volume of 20 µL. The thermal cycler was set as follows: 9′ at 95 °C followed by 30 cycles consisting of 30″ melting at 95 °C + 30″ annealing at 60 °C + 35″ extension at 72 °C. GAPDH expression was used as the housekeeping control for normalization. The comparative CT method (2^-∆∆ct^) was used to quantify gene expression. Melting curve analysis was performed to ensure all transcripts under investigation would be represented by a single peak, as an index for specificity (melting ramp from 70 to 95 °C). *PAX6* [44] and *NESTIN* [55] expression relative to *GAPDH* was performed by using the following primers:

PAX6_FW_GTCCATCTTTGCTTGGGAAA

PAX6_RV_TAGCCAGGTTGCGAAGAACT

NESTIN_FW_ GCTCAGGTCCTGGAAGGTC

NESTIN_RV_TAAGAAAGGCTGGCACAGGT

GAPDH_FW_ACGAATTTGGCTACAGCAACAGGG

GAPDH_RV_TCTACATGGCAACTGTGAGGAGG

### 4.11. Statistical Analysis

Data are presented as a Mean ± Standard Deviation (Mean ± SD) unless otherwise noted. Cytotoxicity, Viability and fusion index data were analysed using one-way ANOVA with Tukey’s correction, as the mean of each dataset was compared to the mean of all the others. Two-way ANOVA with Dunnet’s correction was used to analyse cell alignment data, as the mean of each dataset was compared to that of the control condition.

## 5. Conclusions

Reliability of scaffold for tissue engineering is an issue that regenerative medicine must address, especially when research innovations are translated to clinical practice. Particularly, they have to combine standardised nanocomposite formulations and ease of fabrication with robust and consistent biological response. In graphene-based nanocomposite scaffolds, issues with biocompatibility may even arise from just using different batches of the same GBN, as flakes dimensionality, type and amount of chemical impurities, may vary due to different purification strategies [13]. In this work, we used a graphene@PLA nanocomposite filament consisting as a matrix of pure PLA, a polymer that is FDA-certified for regenerative medicine, and, as nanofiller, graphene G+, which is produced by means of a physical method ensuring the absence of any chemical contaminant. Even though the absence of cytotoxicity for neuronal-like cells and other cellular types of carbon-based nanomaterials dispersed in PLA was already established by several works, hand-made fabrication of such nanocomposite scaffolds may result in unpredictable influence on cell fate and differentiation [40]. Whilst retaining the biocompatibility feature of pure PLA as a matrix and conductivity feature of pristine graphene as nanofiller, FDM 3D-printing with the graphene@PLA and pure PLA filaments used in this work also excluded hand-made batch variation effects on cell growth and differentiation. Furthermore, 3D-printing allowed at the same time to investigate the effect of topographic patterning of scaffolds on cell orientation. In this pilot study, we exploited the effect of scaffolds with different topography and composition on growth, commitment and/or differentiation of iPSCs, neuronal-like cells, immortalised fibroblasts, and primary myoblasts, demonstrating that the specific scaffold topography can promote cell alignment, whereas the presence of graphene promoted iPSC commitment to neuroectoderm and myoblast fusion into multinuclear myotubes.

## Supplementary Materials

The following supporting information can be downloaded at: https://www.mdpi.com/article/10.3390/ijms23031736/s1.
