# Supplementary material for: 3D Printed Graphene-PLA Scaffolds Promote Cell Alignment and Differentiation
Source: Int J Mol Sci. 2022 Feb 3;23(3):1736. doi: 10.3390/ijms23031736 (PMC8836229; doi:10.3390/ijms23031736)
Supplement: Supplementary file 1 [file ijms-23-01736-s001.zip › ijms-1532414-supplementary.pdf]

Supplementary information to manuscript:

3D Printed Graphene-PLA Scaffolds Promote Cell Alignment and Differentiation

Matteo Gasparotto [1,†], Pietro Bellet [1,†], Giorgia Scapin [2,*], Rebecca Busetto [1], Chiara Rampazzo [3], Libero Vitiello [3,4,5], Dhvanit Indravadan Shah [2] and Francesco Filippini [1,*]

1 Synthetic Biology and Biotechnology unit, Department of Biology, University of Padua, 35131 Padua, Italy, matteo.gasparotto.1@phd.unipd.it (M.G.); pietro.bellet@studenti.unipd.it (P.B.); rebecca.busetto@studenti.unipd.it (R.B.)
2 Garuda Therapeutics, Cambridge, MA, 02142, USA; dhvanit.shah@garudatx.com
3 Department of Biology, University of Padua, 35131 Padua, Italy; chiara.rampazzo.1@unipd.it (C.R.), libero.vitiello@unipd.it (L.V)
4 Interuniversity Institute of Myology (IIM), Italy.
5 Inter-Departmental Research Center for Myology (CIR-Myo), University of Padua, 35131 Padua, Italy.
* Correspondence: giorgia.scapin@garudatx.com (G.S.); francesco.filippini@unipd.it (F.F.)
† These authors contributed equally to this work.

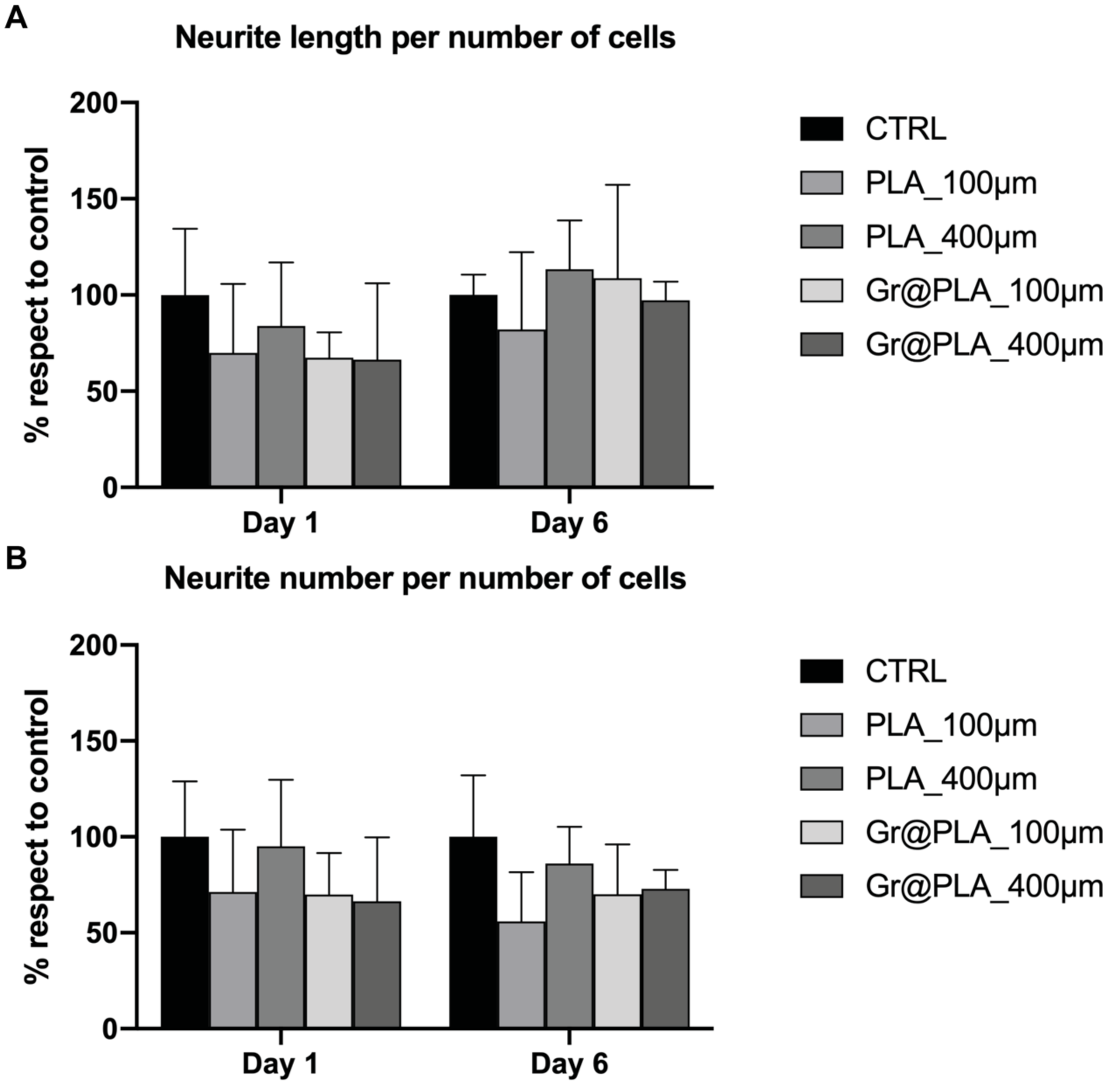

Supplementary Figure S1 – Scaffold induced neuritogenesis of SH-SY5Y cells. (A) Total neurite length per number of cells and (B) total neurite number per number of cells. Cell growth onto scaffolds do not significantly increase neuritogenesis levels respect to cells seeded on plastic slides used as controls.
